# Patterns and mechanisms of major trauma injuries during and after the UK Covid-19 Nationwide lockdown: analysis from a UK Major Trauma Centre

**DOI:** 10.1007/s00068-022-01964-5

**Published:** 2022-05-18

**Authors:** Alfred Adiamah, Christopher Lewis-Lloyd, Jaspreet K. Seehra, Adil Rashid, Edward Dickson, Nick Moody, Lauren Blackburn, John-Joe Reilly, John Saunders, Adam Brooks, ICON Trauma Study Group, Alfred Adiamah, Fady Anis, Ruth Anogo, James Bennett, Lauren Blackburn, Adam Brooks, Rachel Brailsford, Atiba Akii Bua, Amanjeet Dahaley, Ketan Dhital, Edward Dickson, Zoe Draper, Ramzi Freij, Wendy Gaskin, Sunil Gida, Michael Hall, Tanvir Hossain, Lauren Hutchinson, Jamaall Jackman, Audrey Kapeleris, Christopher Lamb, Christopher Lewis-Lloyd, Angelo La Valle, Shane McSweeny, Yasar Nassif, Alex Navarro, Ciara O’Sullivan, Rory O’Connor, Olamide Oyende, Adil Rashid, Melroy Rasquinha, John-Joe Reilly, Sabrina Samuels, John Saunders, Jaspreet Seehra, Bhairavi Srikumar, Laura Sandland Taylor, Melissa Shaw, Vei Lynn Tay, Amari Thompson, Elena Theophilidou, Sue Tumilty, Benjamin Varghese, Robert Winter

**Affiliations:** East Midlands Major Trauma Centre, Nottingham University Hospitals NHS Trust and University of Nottingham, Queen’s Medical Centre, Nottingham, NG7 2UH UK

**Keywords:** Major trauma, Covid-19, Injury severity, Mortality, ICON-TRAUMA

## Abstract

**Purpose:**

To compare patterns and mechanisms of injuries during and after the UK Nationwide lockdown during the COVID-19 pandemic.

**Methods:**

This prospective cohort study included all major trauma admissions during the 10-week period of the nationwide lockdown (09/03/2020–18/05/2020), compared with admissions in the 10-weeks following the full lifting of lockdown restrictions (04/07/20–12/09/2020). Differences in the volume, spectrum and mechanism of injuries presenting during and post-lockdown were compared using Fisher’s exact and Chi-squared tests as appropriate. The associated risk of 30-day mortality was examined using univariable and multivariable logistic regression.

**Results:**

A total of 692 major trauma admissions were included in this analysis. Of these, 237 patients were admitted during the lockdown and 455 patients were admitted post-lockdown. This represented a twofold increase in trauma admission between the two periods. Characteristically, both cohorts had a higher proportion of male patients (73.84% male during lockdown and 72.5% male post-lockdown). There was a noted shift in age groups between both cohorts with an overall more elderly population during lockdown (*p* = 0.0292), There was a significant difference in mechanisms of injury between the two cohorts. The 3-commonest mechanisms during the lockdown period were: Road traffic accidents (RTA)—31.22%, Falls of less than 2 m—26.58%, and falls greater than 2 m causing 22.78% of major trauma admissions. However, in the post-lockdown period RTAs represented 46.15% of all trauma admissions with falls greater than 2 m causing 17.80% and falls less than 2 m causing 15.16% of major trauma injuries. With falls in the elderly associated with an increased risk of mortality. In terms of absolute numbers, there was a twofold increase in major trauma injuries due to stabbings and shootings, rising from 25 admitted patients during the lockdown to 53 admitted patients post-lockdown.

**Conclusions:**

The lifting of lockdown restrictions resulted in a twofold increase in major trauma admissions that was also associated with significant changes in both the demographic and patterns of injuries with RTA’s contributing almost half of all injury presentations.

**Trial registration:** This study was classed as a service evaluation and registered with the local audit department, registration number: 20-177C.

## Introduction

The seasonal variability in major trauma injuries and presentation is a well-known phenomenon [1]. However, the COVID-19 pandemic and the associated UK nationwide lockdown led to an unprecedented change in societal behaviours and opportunities for harm that ultimately led to the well-reported decreases in admissions of major injured patients across most of the westernised societies [2]. Whilst some reported an associated alteration in injury severity and mechanism of injuries, such as an increase in low falls in the older adults, others found no such differences. Nevertheless, this new variation in patterns and demographics of injuries seen was expected to be temporary and a direct consequence of the pandemic.

On the 4th of July, 2020, the nationwide lockdown measures were fully lifted in England, and a return to normality ensued with an expected upsurge in road transport, along with the opening of public houses and workspaces previously deemed unessential [3]. The opportunities to engage in activities socially and behaviours known to be associated with interpersonal violence were also expected to rise. However, whether this expected rise would result in a rebound effect or further alterations to the demographics, patterns and severity of major trauma admissions is not known and has not been studied.

During the lockdown, most hospital services including the major trauma service, adapted to the COVID-19 pandemic by reallocating resources, redistributing the workforce and optimising service provision. Post-lockdown, these adaptations require re-evaluation to account for any changes in the frequency, urgency and injury severity of the major trauma injured patient. Whilst a prior study had compared the effects of the pandemic lockdown to a pre-lockdown period in 2019 [2], this follow-on study evaluated the post-lockdown period. It aimed to identify the changes in demographics, patterns, severity and mechanisms of injury during the post-lockdown period to understand the burden of traumatic injuries on patients and on the Major Trauma service.

## Methods

This prospective observational study was undertaken at the East Midlands Major Trauma Centre (EM-MTC). This provides regional major trauma cover for a population of 3.8 million. A 10-week period between the 9th of March and 18th of May 2020, defined as the “lockdown period” was compared with the “post-lockdown period”. The latter was defined as the 10-weeks following the full lifting of the nationwide lockdown from the 4th of July to the 12th of September 2020.

### Inclusion

All Major Trauma patients admitted during the lockdown and post-lockdown periods were included. Major trauma was defined using UK NICE guidelines definition, that has been used in previous studies and is accepted by all UK national trauma centres as an injury or combination of injuries that are life threatening and could be life changing because it may result in long-term disability [2].

### Primary outcomes

To compare the weekly frequency of trauma admissions during and post-lockdown, against the previously established estimate of 30-40 in-patient admissions per week.

### Secondary outcomes

The demographic differences, mechanism of injuries, length of stay, management approach and 30-day mortality were evaluated.

### Definitions of covariates

Age at the time of admission was categorised into 0–17, 18–39, 40–64 and ≥65 years. Gender was coded as male or female. Clinical frailty was calculated using the Rockwood clinical frailty scale (CFS) [4] and patients categorised into one of three distinct groups: Non frail (CFS 1–3), vulnerable to mildly frail (CFS 4–5) and moderate to severely frail (CFS 6–9). Comorbidity was defined using the Charlson co-morbidity index and split into four categories, each with an associated percentage 10-year survival estimation [5]. Socioeconomic information was defined into quintiles from the English Index of Multiple Deprivation (IMD) 2019 [6]. Ethnicity was determined from data already held in hospital records and defined using nationally agreed guidelines as Asian, Black, Mixed, Other and White.

Injury severity score (ISS) was defined as minor and major trauma based on ISS ≤ 15 and ≥ 16, respectively. [7]. Mechanism of injury was classified into 9 categories as Blows, Burns, Crush, Fall < 2 m, Fall > 2 m, Shooting/stabbing, Vehicle Incident/Collision and Other. A SARS-CoV-2 diagnosis was established by either a positive PCR (reverse transcriptase-polymerase chain reaction) swab result or based on clinical and radiological features of SARS-CoV-2 pneumonitis on CT thorax within 30 days of admission. Management on admission was defined as: conservative, interventional radiological or surgical.

### Statistical analysis

The basic demographic characteristics of the lockdown and post-lockdown cohorts are described using frequencies and percentages for categorical variables and medians and interquartile ranges (IQR) for continuous, with Fisher’s exact, Chi-squared and Mann–Whitney *U* tests used for significance testing as appropriate. The crude 30-day mortality in both cohorts was assessed by age, gender, mechanism of injury and frailty. Univariable and multivariable logistic regression models were used to define the factors associated with an increased risk of 30-day mortality. To account for any changes in trauma admissions, a three year analysis of weekly trauma admissions between 2017 and 2019 was undertaken to establish a baseline for comparison. All data were analysed using STATA V16 (StataCorp, Stata Statistical Software: Release 16, College Station, Texas, UK). Statistical significance was set at the 95% level and a p-value of less than 0.05 considered statistically significant.

### Ethics and consent

The study was registered and approved locally by the institutional review boards as a service evaluation, registration number 20-177C. Individual patient consent was waived.

## Results

### Demographics

A total of 692 major trauma admissions were included in this analysis. Of these, 237 patients were admitted during the lockdown period and 455 patients admitted in the post-lockdown period. This represented a twofold increase in trauma admission between the two periods. Characteristically, both cohorts had a higher proportion of male patients (73.8% male in lockdown, 72.5% male post-lockdown). There was also an increase in the proportion of younger patients in the post-lockdown period. The number of under-40 s rose as a percentage of all admissions from 43.5% to 51.8%. The post-lockdown period also saw a fall in the number of patients aged over 65 years, from 20.3% to 16.5% (*p* = 0.0292). However, the post-lockdown cohort had a higher proportion of moderately to severely frail patients (lockdown 11.39% vs post-lockdown 22.86%, *p* = 0.0003) (Table 1).

**Table 1:** Cohort demographics and characteristics of COVID-19 lockdown and post lockdown periods

(*n* = 692)					
	Lockdown (*n* = 237)		Post lockdown (*n* = 455)		*p*-value^a^
Age (years)					
0–17	16	6.75%	47	10.33%	0.0292^b^
18–39	87	36.71%	189	41.54%	
40–64	86	36.29%	144	31.65%	
≥65	48	20.25%	75	16.48%	
Sex					
Female	62	26.16%	125	27.47%	0.7124
Male	175	73.84%	330	72.53%	
Rockwood Clinical Frailty Scale					
Non frail (1–3)	197	83.12%	324	71.21%	0.0003^b^
Vulnerable to Mildly frail (4–5)	13	5.49%	27	5.93%	
Moderate to Severely frail (6–9)	27	11.39%	104	22.86%	
Charlson Comorbidity Index					
0 (98% 10-year survival)	134	56.54%	297	65.27%	0.0050
1–2 (≥90% 10-year survival)	55	23.21%	74	16.26%	
3–4 (>50% 10-year survival)	23	9.70%	59	12.97%	
≥5 (<25% 10-year survival)	25	10.55%	25	5.49%	
Ethnicity					
Asian	7	2.95%	20	4.40%	0.7369
Black	6	2.53%	8	1.76%	
Mixed	2	0.84%	7	1.54%	
Other	20	8.44%	41	9.01%	
White	202	85.23%	379	83.30%	
Socioeconomic status					
1 (most deprived)	47	19.83%	99	21.76%	0.6103^b^
2	52	21.94%	91	20.00%	
3	44	18.57%	80	17.58%	
4	41	17.30%	86	18.90%	
5 (least deprived)	52	21.94%	93	20.44%	
Missing	1	0.42%	6	1.32%	
Trauma call code					
Green/Amber	226	95.36%	408	89.67%	0.0104
Red	11	4.64%	47	10.33%	
Time to CT Scan in minutes					
Time from CT decision to CT Scan (Median (IQR))^e^	32	17–75	30	19–89	0.5662^c^
Time from ED arrival to CT Scan (Median (IQR))^f^	42	24–75	40	23–80	0.5577^c^
Mechanism of injury					
Blows	5	2.11%	13	2.86%	0.0003
Burn	5	2.11%	3	0.66%	
Crush	3	1.27%	2	0.44%	
Fall < 2 m	63	26.58%	69	15.16%	
Fall > 2 m	54	22.78%	81	17.80%	
Other	7	2.95%	23	5.05%	
Stabbing/Shooting^g^	26	10.97%	54	11.87%	
Vehicle Incident/Collision	74	31.22%	210	46.15%	
ISS					
0–15 (Minor Trauma)	177	74.68%	376	82.64%	0.0133
≥16 (Major Trauma)	60	25.32%	79	17.36%	
Definitive management					
Conservative	175	73.84%	310	68.13%	0.0570
Interventional radiology	0	0.00%	8	1.76%	
Surgical	62^ h^	26.16%	137	30.11%	
COVID-19 diagnosis					
Negative	233	98.31%	455	100.00%	0.014^d^
Positive	4	1.69%	0	0.00%	
30-day mortality					
No	214	90.30%	429	94.29%	0.0523
Yes	23	9.70%	26	5.71%	

*ISS* Injury Severity Score, *CT* Computed Tomography, *ED* Emergency Department, *IQR* Interquartile range

^a^Chi-Square (*χ*2) Test

^b^Chi-Square (*χ*^2^) Test for trend

^c^Mann Whitney *U* Test

^d^Fisher's Exact Test

^e^Lockdown *n* = 117, Post Lockdown *n* = 343

^f^Lockdown *n* = 151, Post Lockdown *n* = 373

^g^Only 1 Shooting within the whole cohort that occurred during the COVID-19 Lockdown period

^h^Includes 1 patient that underwent interventional radiological embolisation prior to surgery during the COVID-19 Lockdown period

### Trauma admissions

**Fig. 1:**
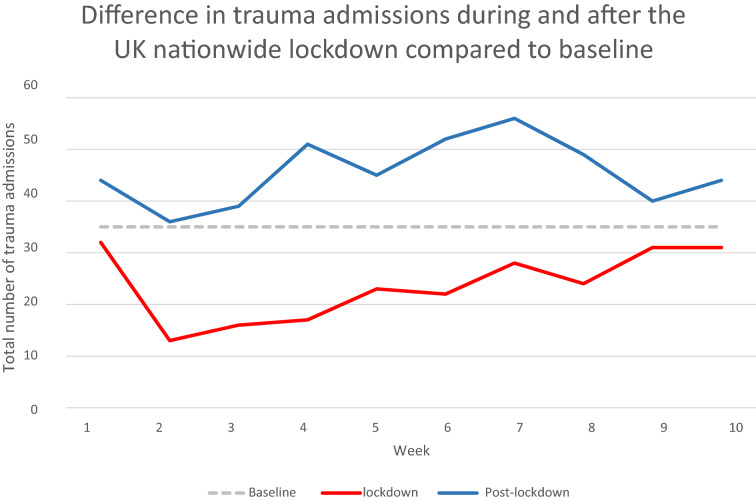
Compared to baseline, there was a 32% reduction in trauma admissions during the 10 week period of the lockdown and a 30% increase in a similar time period post-lockdown. However, comparing lockdown to post-lockdown there was a 91% increase in a total number of patients admitted over that 10-week block

The baseline count of trauma admission over the 3-year period (2017–2019) was a mean of 35 (SD ± 2) patients per week. Compared to this baseline, there was an overall 32.3% decrease in trauma admissions during lockdown (237 instead of an expected 350 over the 10-week period). Contrastingly post-lockdown period there was a 30% increase above this baseline (Fig. 1). Code red trauma patients are those deemed to be haemodynamically unstable pre-hospital and/or those who require activation of major haemorrhage protocol. The percentage of patients admitted as a code red rose from 4.64 to 10.33% between the lockdown and post-lockdown cohorts, respectively (*p* = 0.0104). This demonstrates a rise in the absolute number of trauma admissions, as well as the severity of injuries sustained in the post-lockdown period (Table 1).

### Mechanism of injury

**Fig. 2:**
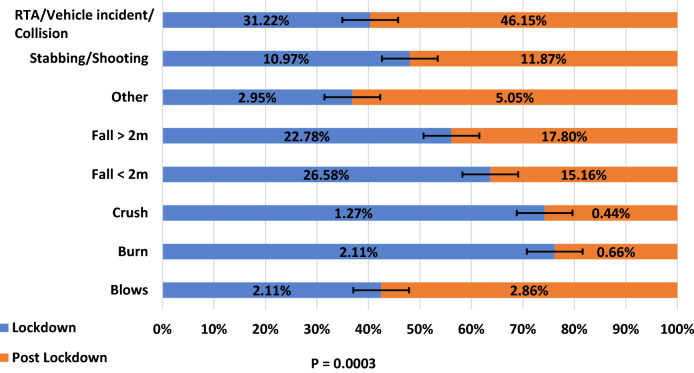
Overall, there were changes in mechanisms of injuries between the lockdown and post-lockdown periods. (*p* = 0.0003). Each bar chart represents a different mechanism of injury in isolation and compared in terms of proportion they contribute to a total number of injuries during and post-lockdown. The mechanisms that change (do not cross the 50% mark) include RTA’s (vehicle incidents and collision), Falls > 2 m, Falls < 2 m, Burns, Crush injuries and Blows

There was a significant difference in the mechanism of injury between the lockdown and post-lockdown cohorts. Whilst the 3 most common mechanisms of injury contributing to trauma admission in both cohorts were RTA, falls greater than 2 m and falls less than 2 m, the proportion each contributed varied significantly between periods. In the post-lockdown period, RTA’s contributed 46.15% of all trauma admissions, up from 31.22%. Falls greater than 2 m contributed 22.78%, down from 26.58% and falls less than 2 m contributed 15.16% down from 26.58%. In terms of absolute numbers, there was a twofold increase in major trauma injuries due to stabbings and shootings, rising from 25 admitted patients during the lockdown to 53 admitted patients post-lockdown. However, as a proportion of the total number of trauma admissions the proportions remained comparable with 10.97% of admissions due to stabbings and shootings during the lockdown and 11.87% of admissions post-lockdown. Figure 2 demonstrates the change in the mechanism of injury during the two periods.

### Severity of injury and mortality

The proportion of major trauma patients with an ISS > 16 was higher in the lockdown cohort compared to the post-lockdown cohort (25.32% vs 17.36%). 30-day mortality was also higher in the lockdown cohort, with 23 deaths (23/237, 9.70%) compared to 26 (26/455, 5.71%) in the post-lockdown cohort.

Factors found to impact mortality were older age, frailty and falls as a mechanism of injury (Table 2). Older and frailer patients had higher mortality regardless of which cohort they belonged to, as did those admitted with falls (Table 2).

**Table 2:** Mortality by age, sex, mechanism of injury and Rockwood clinical frailty scale

`	Lockdown				Post lockdown			
	Total	Number of deaths (%)		*p*-value^a^	Total	Number of deaths (%)		*p*-value^a^
Age (years)								
0–17	16	1	6.25	<0.0001^b^	47	0	0.00	<0.0001^b^
18–39	87	0	0.00		189	4	2.12	
40–64	86	7	8.14		144	8	5.56	
≥65	48	15	31.25		75	14	18.67	
Sex								
Female	62	10	16.13	0.0472	125	11	8.80	0.0813
Male	175	13	7.43		330	15	4.55	
Mechanism of injury								
Blows	5	0	0.00	0.0494	13	1	7.69	0.0029
Burn	5	0	0.00		3	0	0.00	
Crush	3	0	0.00		2	0	0.00	
Fall < 2 m	63	8	12.70		69	12	17.39	
Fall > 2 m	54	11	20.37		81	4	4.94	
Other	7	0	0.00		23	1	4.35	
Stabbing/shooting	26	0	0.00		54	1^c^	1.85	
Vehicle incident/collision	74	4	5.41		210	7	3.33	
Rockwood Clinical Frailty Scale								
Non frail (1–3)	197	7	3.55	<0.0001^b^	324	10	3.09	0.0005^b^
Vulnerable to Mildly frail (4–5)	13	4	30.77		27	5	18.52	
Moderate to Severely frail (6–9)	27	12	44.44		104	11	10.58	
Total	237	23	9.70	–	455	26	5.71	–

Percentages are row percentages

^a^Chi-Square (*χ*2) Test

^b^Chi-Square (*χ*2) Test for trend

^c^Death was from a Stabbing injury

#### Management

There was no statistical difference in the definitive management of major trauma patients during and post-lockdown. Conservative and surgical treatment was employed in 73% and 26% of patients during the lockdown and 68% and 30% of patients’ post-lockdown respectively (*p* = 0.0570) (Table 1).

## Discussion

This study demonstrated that whilst major trauma admissions fell during the lockdown period, there was a large increase in admissions upon lifting of restrictions, with a rebound effect amounting to a 91% difference in the number of admissions during and post-lockdown. This rise in admissions was 30% higher than would be expected in a usual 10-week period. Given one of the main adaptations during lockdown was a streamlining of staff, this finding of an almost twofold increase in trauma admissions post-lockdown is important for planning and service provision.

Falls from greater than 2 m height and RTA’s were the greatest contributors to this rebound effect, increasing by 50 and 184% respectively. This suggests a return to the baseline level of activities, with possibly an increase in risk-taking behaviour explaining the rise in the number of people falling from a greater than 2 m height.

Patients presenting post-lockdown were also more likely to be classed as red trauma calls due to prehospital haemodynamic instability, however, following in-hospital assessment patients during the lockdown period were more likely to have higher ISS. This is difficult to account for. It may reflect a disinclination of pre-hospital practitioners to activate code-red trauma calls during the lockdown period, possibly due to an awareness that hospitals are under COVID-related pressure. Alternatively, this may be due to pre-hospital practitioners with greater experience continuing in their role, whilst those with less experience may have re-deployed to the hospital during the lockdown period, resulting in a skewing of these results. If such results are replicated elsewhere, consideration should be given to assessing for such factors.

During the lockdown, there was a more elderly and more comorbid population compared to a younger and less comorbid cohort post-lockdown. Interestingly, the lockdown did not affect the management strategies utilised.

As the first Western country to be severely hit by the COVID-19 pandemic, Italian centres anecdotally observed an estimated 50% decrease in trauma volume with an increasing severity in the presentation of major trauma injuries [8]. Nunez et al. similarly found a decreasing frequency of trauma presentations from workplace accidents and RTA’s at a Spanish Tertiary Trauma Centre after the Spanish state of emergency was declared [9]. Within the USA, a study from New Jersey also observed a reduction in trauma admissions, with less RTCs and being struck by objects [10]. Interestingly, they noted a 28.9% decrease in falls [10]. The Californian state put a ‘shelter-in-place’ order to improve social distancing and reduce virus transmission and noticed a 4.8 fold reduction in trauma activations [11]. In the UK, orthopaedic trauma admissions dropped in both adult and paediatric populations [12]. A different UK centre observed similar results with no changes to the mechanism of injury [13]. A German study observed a decrease in most trauma mechanisms and injury patterns during the lockdown. (1) Our findings are concurrent with these studies that showed a decrease in trauma admissions during the lockdown period. However, ours found both a more severely injured patient in terms of ISS scores and an altered mechanism of injury. Additionally, our study has shown that following lockdown, there is a significant increase in trauma admissions causing a rebound effect above the expected caseload.

The demographic characteristics of the major trauma injured patient changed during the lockdown, and importantly, this change was associated with an increased mortality risk. A study using data from Trauma and Audit Research Network (TARN) [14] highlighted the elderly and frail as a vulnerable group of patients who harbour a higher risk of mortality, even following falls from standing (falls less than 2 m) [15]. There was a significant difference in the proportion of frail patients admitted, with those with a CFS of 5–9 making up 11% of admissions during the lockdown and 23% post-lockdown. Mortality in this group however was lower in the post-lockdown admissions. This may relate to factors outwith their clinical condition, such as factors related to wider hospital system processes in the midst of lockdown. In both the lockdown and post-lockdown periods, elderly age and frailty were the patient factors most associated with an increased mortality risk. Thompson et al., have previously demonstrated the importance of frailty (defined using the clinical frailty score) in prognosticating major trauma injured patients.

Alternatively, it may be that the rise in admissions, predominantly those occurring as a result of RTA’s, are not associated with mortality. The increasing safety of motor vehicles has been well documented, with UK statistics in 2019 showing a 21% fall in fatalities since 2009 [16].

RTA in adults are the leading cause of trauma admissions in the UK and this was also found to be true both during and after the lockdown. However, post-lockdown with the lifting of the laws enforcing only “essential travel” we found RTAs accounted for almost 50% of all trauma admissions. Driving surveys also suggested a sharp increase in RTAs after lifting of the lockdown by an estimated 72% [17]. During the lockdown, the UK Department of Transport issued an MOT extension to ensure keyworkers could maintain mobility without risking opening MOT centres. There are suggestions that this could have led to a surge in unsafe vehicles on the road after lockdown and could in part account for some of the rise in RTA’s seen [18].

Within South Africa, Morris et al. identified a reduction in life-threatening injuries secondary to interpersonal violence (gunshot wounds and knife injuries) suggesting that level 5 lockdown in the province of Kwa-Zulu Natal had an unexpected positive result [19]. However, despite this decrease, they noted that severity remained unchanged [19]. Our study found a twofold increase in major trauma injuries due to stabbings and shootings, rising from 25 admitted patients during the lockdown to 53 admitted patients post-lockdown. Suggesting that within our population the lockdown had a similar effect as found in the South African study. Across England and Wales, the office of national statistics also reported a drop-in knife crime by 1% during the pandemic [20].

The initial ICON trauma study [2], highlighted the shift in mechanism of injury to falls less than 2 m in a predominantly elderly and vulnerable population. It has been reported that about 26 and 17% of elderly people who required assistance with 1 and 2 activities of daily living (ADL) prior to the pandemic went on to receive no help during the lockdown. This could have contributed to an increased risk of injury. By contrast, in this post-lockdown cohort, the proportion of trauma admissions with falls declined which could be due to reinstating of social support and family support networks [2]. Although this association has not been studied directly, family and social support systems during the pandemic were found to be protective of the mental, and physical wellbeing of the elderly and groups classed as vulnerable [21].

## Limitations

This is one of the largest studies to investigate the post-lockdown effect in trauma patients and has demonstrated a significant rebound phenomenon in number of admitted patients. It has also shown a change in injury characteristics and demographics. However, it is a single centre study and whilst data collection was prospective it remains at risk of bias usually associated with observational studies. However, we have sought to reduce selection bias by including “all patients” admitted prospectively during these defined cohorts. The time periods used, were linked to the dates during and after the UK lifted restrictions, which may not fully account for the seasonal variability in trauma admissions. Additionally, the population characteristics of our region might not be fully representative of the entire UK population and a multi-centre study of this kind would provide added insight into the changes in the mechanism and demographics by region. A further limitation is the inability to comment on the underlying cause of injury such as deliberate self-harm/attempted suicide which may influence the mechanisms of trauma and mortality observed. This is because accurate identification of such causes is often difficult within a trauma setting and may not be recorded within hospital notes and could therefore represent an underreporting of cases.

## Conclusion

This study found that following the lifting of lockdown restrictions in the UK, trauma admissions rebounded, with an increase in overall admissions, and a reversion in the demographics to a predominantly younger male population. The main mechanism of injury also differed between the two cohorts, from falls as the predominant injury mechanism during lockdown to a surge in RTAs post-lockdown. Future lockdowns should consider prioritisation of community social care resource allocation to ensure a reduction in falls in an isolated and increasingly elderly population. Whereas post-lockdown periods will require a focus on road safety and other risk-taking behaviour.

## Data Availability

Data available on request via corresponding author.
